# Environmental Enrichment Expedites Acquisition and Improves Flexibility on a Temporal Sequencing Task in Mice

**DOI:** 10.3389/fnbeh.2018.00051

**Published:** 2018-03-15

**Authors:** Darius Rountree-Harrison, Thomas J. Burton, Catherine A. Leamey, Atomu Sawatari

**Affiliations:** ^1^Discipline of Physiology, School of Medical Sciences and the Bosch Institute, University of Sydney, Sydney, NSW, Australia; ^2^Animal Behavioural Facility, School of Medical Sciences and the Bosch Institute, University of Sydney, Sydney, NSW, Australia

**Keywords:** environmental enrichment, learning, memory, cognitive flexibility, temporal order, mice, behaviour

## Abstract

Environmental enrichment (EE) via increased opportunities for voluntary exercise, sensory stimulation and social interaction, can enhance the function of and behaviours regulated by cognitive circuits. Little is known, however, as to how this intervention affects performance on complex tasks that engage multiple, definable learning and memory systems. Accordingly, we utilised the Olfactory Temporal Order Discrimination (OTOD) task which requires animals to recall and report sequence information about a series of recently encountered olfactory stimuli. This approach allowed us to compare animals raised in either enriched or standard laboratory housing conditions on a number of measures, including the acquisition of a complex discrimination task, temporal sequence recall accuracy (i.e., the ability to accurately recall a sequences of events) and acuity (i.e., the ability to resolve past events that occurred in close temporal proximity), as well as cognitive flexibility tested in the style of a rule reversal and an Intra-Dimensional Shift (IDS). We found that enrichment accelerated the acquisition of the temporal order discrimination task, although neither accuracy nor acuity was affected at asymptotic performance levels. Further, while a subtle enhancement of overall performance was detected for both rule reversal and IDS versions of the task, accelerated performance recovery could only be attributed to the shift-like contingency change. These findings suggest that EE can affect specific elements of complex, multi-faceted cognitive processes.

## Introduction

The ability to quickly formulate a consistent course of action based on salient events, as well as rapidly adjust behaviours to abrupt changes in contingencies is vital for survival. Often, relevant events happen in quick succession and thus require the ability to temporally resolve experiences. Further, the chronological order of these past events in and of themselves can provide vital information for choosing a correct series of actions.

The acquisition of stable rules (Wallis et al., 2001) and schemas (Tse et al., 2011) that map recently experienced event sequences and actions to desirable outcomes (Takahashi et al., 2011; Wilson et al., 2014), as well as the ability to then subsequently adjust behaviours to changing contingencies, have been shown to engage dissociable learning and memory processes (Lee et al., 2015). Each of these processes are regulated by a series of interconnected brain regions (Packard et al., 1989). Mnemonic storage and recall of both recently and distally experienced past events are associated with interplay between the hippocampal formation and prefrontal circuits (Spellman et al., 2015). The ability to use this temporal information to guide choices that ultimately lead to positive outcomes in turn require the coordinated activation of hippocampal, prefrontal and striatal networks (Tse et al., 2011; Tsujimoto et al., 2011; Rossi et al., 2012; Gourley et al., 2013, 2016; Sleezer et al., 2016).

Environmental enrichment (EE), which exposes subjects to enhanced levels of sensory stimuli, social interaction, and/or voluntary exercise (Hebb, 1947), has been shown to dramatically affect both the development and function of many of the circuits involved in sequence memory storage and retrieval (Donato et al., 2013), rule acquisition (Harburger et al., 2007), as well as cognitive flexibility (Simonetti et al., 2009; Zeleznikow-Johnston et al., 2017). EE can accelerate the maturation of developing striatal circuits (Simonetti et al., 2009), increase neurogenesis (Kempermann et al., 1997), and promote dendritic/axonal arborisation and synaptogenesis in the hippocampus (Donato et al., 2013, 2015) as well as cortex (Gelfo et al., 2009; Jung and Herms, 2014).

Behaviourally, EE has been shown to influence spatial memory acquisition and recall (Garthe et al., 2016; Hüttenrauch et al., 2016), olfactory recognition and discrimination (Mandairon et al., 2006a,b,c; Weeden et al., 2014), both reliant on hippocampal function, as well as reversal learning in a visual discrimination task (Zeleznikow-Johnston et al., 2017), a process revealed to be dependent on the striatum and prefrontal areas (Gourley et al., 2013, 2016). The manner in which EE affects the acquisition and performance of tasks that explicitly incorporate elements of reference and working memory, as well as cognitive flexibility has not been well characterised.

As a first step, an established memory assay, the Olfactory Temporal Order Discrimination (OTOD; Fortin et al., 2002) task, was adapted to examine if and how EE can influence an animal’s ability to acquire and subsequently change appropriate choice behaviours regarding the presentation order of two olfactory exemplars recently experienced in a five odour sequence. The temporal span or distance between choice odours was varied to further determine whether enrichment affected the ability to temporally resolve past events. As mice are heavily reliant on olfaction for food seeking, social interactions, as well as predator avoidance (Perry et al., 2016; Liu et al., 2017), assessment of this sensory modality provides an ideal means to explore naturalistic behaviour.

We found that EE improved acquisition of the OTOD task for all three temporal separations tested. Change point analyses on cumulative performance profiles of individual animals revealed that EE was associated with a significantly earlier upward transition in performance, particularly for middle and long intervals, indicating an expedited learning of task rules in these mice. Enriched groups also exhibited small but detectable performance improvements in two different versions of the same task with specifically altered task contingencies.

Together, these findings suggest that EE from birth can affect the use of multiple memory systems to better adapt to a rapidly and continuously changing environment.

## Materials and Methods

This study was carried out in accordance with the recommendations of the Australian code for the care and use of animals for scientific purposes (editions 7/8), National Health and Medical Research Council (NHMRC) Guidelines, Animal Welfare Committee. The protocol was approved by Animal Ethics Committee (AEC) of the University of Sydney. All animals were housed in a single room at the University of Sydney Animal Housing Facility on a fixed 12/12 h light/dark cycle. All mice were housed in open-top cages and had *ad libitum* access to food and water, except where specified (see below).

### Environmental Enrichment

**Figure 1:**
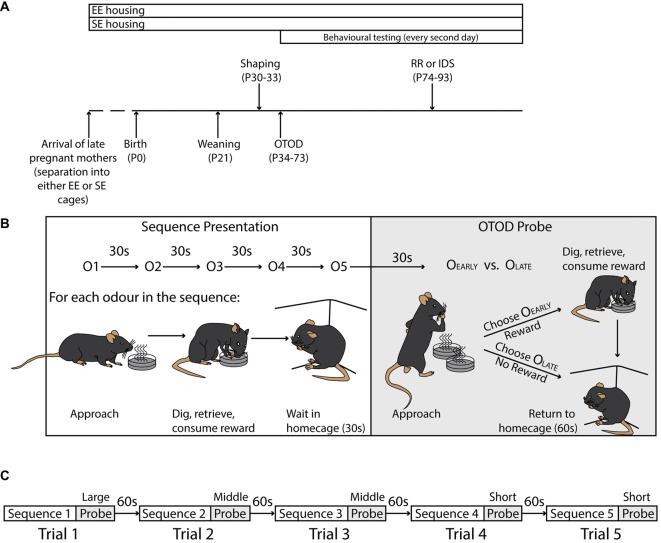
Experimental timeline and task structure. **(A)** Timeline illustrating duration of enrichment (top), and significant experimental time points (bottom). Shaping was initiated at P30, while formal training on the Olfactory Temporal Order Discrimination (OTOD) task began 4 days later. After the initial acquisition period, a subset of each housing cohort was trained on one of two adjusted versions of the task, a version with new olfactory cues, but with the same rule (IDS), or one using the same odours but requiring a different rule (RR). See “Materials and Methods” section for details. **(B)** Schematic illustrating the structure of a single trial including the sequence presentation (left) and OTOD probe (right). Upper portion depicts the presentation of five single odours in sequence (inter-odour interval: 30 s; odour order varied across trials; see “Materials and Methods” section) followed by a probe which consisted of a single simultaneous presentation of two odours. The lower left portion schematically shows the substructure of each odour presentation. For each odour in the sequence, individual mice had to approach the cup, dig in the sand to retrieve the single sunflower seed, and then consume the reward. The subject was then removed from the testing arena and placed in the home-cage for 30 s before the process was repeated for the next odour in the sequence. During the OTOD probe (lower right) animals were presented with two cups containing odours from the sequence, only one of which contained a reward. A correct choice (O_Early_; the earlier of the two odours presented in the sequence), meant that mice were allowed to dig, retrieve and consume a reward of four seeds, before being removed from the testing arena and placed in their home-cage for 60 s before the commencement of the next trial. If an incorrect choice was made (O_Late_), the trial was aborted and subjects were immediately removed from the arena and returned to their home-cage for the 60 s inter-trial interval. All mice were given a total of 90 s to choose, and if successful, consume reward (see “Materials and Methods” section). **(C)** Flowchart detailing session structure in a single training day. Each subject were given five consecutive trials at three different sequence spans (1 × Long, 2 × Middle, and 2 × Short; see “Materials and Methods” section) separated by an inter-trial interval of 60 s (see “Materials and Methods” section).

On arrival into our mouse colony, late pregnant C57BL6 mouse mothers (ARC, Western Australia) were randomly allocated into either enriched environment (EE), or standard environment (SE) housing conditions (see below). All experiments were performed on the offspring of these dams (Figure 1A).

SE cages (12.5 × 30 × 11.5 cm) contained a red plastic igloo, paper chip pelleted bedding, and tissue for nesting. A single pregnant dam was placed in a given SE box.

EE cages were larger (12.5 × 45 × 30 cm) and, in addition to the components found in SE cages, featured a running wheel and a range of sensory stimuli including (but not limited to) cardboard tubing, half tissue boxes, rubber balls, scented cotton balls (e.g., artificial strawberry, vanilla and lemon scents), Velcro strips (adhered to cage walls to provide tactile stimuli), high contrast visual gratings, bell balls etc. These items were rearranged within the cage every second day. Further, late pregnant dams were pair housed in EE boxes to augment social interaction. Access to food and water was identical between mice placed in standard and enriched environments.

Litters remained in the same housing as their dams until weaning. At postnatal day (P) 21, the juveniles were placed into new cages maintaining their EE (23 mice from 2 litters: 5–8 animals per cage) and SE conditions (26 mice from 2 litters: 3–5 animals per cage). Behavioural testing commenced at P30 (Figure 1A).

### Behavioural Testing

On the evening prior to each behavioural session, food pellets were removed to encourage reward seeking behaviour during training/testing. Behavioural sessions took place every second day. Sunflower seeds were used as reward. Animals had *ad libitum* access to food after the completion of each testing/training session.

#### Shaping

To evaluate memory for temporal order of a sequence of events, we exploited the natural proclivities that mice possess for digging and their aptitude for detecting and discriminating olfactory stimuli. Animals were first habituated to a testing arena (arena size: 60 × 60 × 40 cm), and subsequently trained to dig in sand-filled plastic cups for a buried sunflower seed reward linked to olfactory cues. A single odourant (0.01% cinnamon by weight mixed in sand) was used for this stage. This stage was repeated over two training days.

Mice were then exposed to four “weighted” sequence presentations in which five cups with different odours (0.01% odourant by weight; dry, powdered odourants included cardamom, clove, coriander, cumin, garlic, ginger, mustard, nutmeg, paprika, parsley, tea and turmeric), placed at one of two “foraging” locations within the arena (two adjacent corners of the enclosure). Odour cups presented earliest in the sequence contained four seeds, decreasing incrementally by a seed each cup, with the final two cups having no seeds in order to emphasise the importance of presentation order. Placement and order of odours presented were varied for each sequence presentation. Mice had 90 s to consume each reward. The subject was then removed from the arena for 30 s before being placed back in the enclosure with a new odour cup. All mice were given four complete sequences of five odours.

Subsequently, mice were presented with a sequence of five weighted (see above) odour cups followed by a choice test in which two of the odour cups (probe cups) from different time points in sequence were placed in both foraging corners. The subject had to choose the odour cup previously presented in the sequence to gain a reward (four seeds). Mice had 90 s to make a choice. The combination of a given five odour sequence with the choice test constituted a single trial. Each animal was exposed to five trials, each with randomised odour order and reward location; once on a span of three odours between choices (first and last odours: long distance), twice on a span of two odours (e.g., first and fourth or second and fifth; middle distance) and twice on a span of one odour (e.g., first and third; short distance). At the end of each trial, the subject was removed from the arena and placed in their home cage for 60 s before beginning the next trial.

This shaping procedure (including foraging, weighted sequence presentation alone, and weighted sequence presentation with choice test) was completed over four training days for each animal (Figure 1A).

#### Olfactory Temporal Order Discrimination (OTOD) Task

Upon completion of shaping, mice were introduced to the actual OTOD task (Figures 1B,C). Subjects were first exposed to a sequence of five cups of sand with olfactory stimuli, each containing a single reward seed. Mice had 90 s to forage and consume each reward from the sand cups before being removed from the arena. A 30 s inter-presentation interval was included between each odour cup presentation. While the identity of olfactory stimuli remained consistent across OTOD task acquisition (cardamom, nutmeg, paprika, tea, and turmeric), the order of the five odours was varied with each sequence presentation (Figure 1B).

After experiencing the five odours, mice were returned to the arena and presented with a choice test: two of the olfactory stimuli in probe cups, only one of which concealed a reward (four seeds). Animals that chose to dig in the cup containing the odour encountered earlier in the sequence were rewarded (correct choice). Foraging in the cup containing the more recently presented olfactory stimuli was deemed an incorrect response (incorrect choice; not rewarded) and the trial was terminated. The two probe cups were placed in the same two foraging corners within the arena used during shaping, with the rewarded cup assigned in a pseudo-random fashion (balanced for both sides) to avoid any spatial biases in choice selection. Placement of a paw within either cup constituted a choice made. On any given choice presentation, mice were allowed 90 s to find and consume the reward. If the subject did not make a choice in the time allotted (non-response), the probe was terminated and the animal removed from the arena (Figure 1B).

As during the shaping period, a trial consisted of one sequence of five odours and the subsequent probe. Any given discrimination problem was characterised by one of three different temporal “distances”: long (three odours apart; L); middle (two odours apart; M); short (one odour apart; S). On a training day (session), each animal received five consecutive trials (1 L, 2 M and 2 S) with an inter-trial interval of 60 s where the order of odor presentation in the sequence was changed (Figure 1C). Each animal was assessed over a total of 100 trials (20 L, 40 M and 40 S) for the acquisition of this task (Figure 1A).

#### Cognitive Flexibility in the Olfactory Temporal Order Discrimination (OTOD) Task

In order to investigate the effect of EE on cognitive/behavioural flexibility in the OTOD task, either reinforcement or stimulus contingencies were changed in the form of a Rule Reversal (RR) or an Intra-Dimensional Shift (IDS) respectively. To be rewarded, RR required animals to adapt their decision-making strategy to an inversion of the previously learned rule (i.e., choosing the odour experienced later in the sequence instead of that which was experienced earlier). The IDS required the animals to apply the learned rule to novel stimuli of the same olfactory modality (i.e., five new exemplar odours were used though the rules of the task did not change; Figure 1A).

A subset of each housing group (EE: *n* = 7 for RR, *n* = 7 for IDS; SE: *n* = 6 for RR, *n* = 6 for IDS) were trained on either one of these two protocols in the same fashion as the initial OTOD for 50 trials (10 L, 20 M and 20 S) to assess the influence of enrichment on task performance immediately after the changed conditions were initiated.

### Analysis

Due to the binary nature of the outcome, i.e., either a “correct” or “incorrect” choice, associations between housing condition, temporal span and performance were evaluated using general estimating equations (GEE; SPSS, IBM Corporation, NY, USA). A binomial distribution with a logit (logarithm of the odds ratio (OR): the probability of choosing to correct choice over the probability of choosing the incorrect choice) link function was used to model task acquisition, RR and IDS versions of OTOD. Associations between housing and performance were also modelled separately for each distance. Outcomes are presented as regression coefficients (b), standard errors in regression estimates (s.e.r.) and OR. OR values greater than one indicate significantly (*α* = 0.05) increased likelihood of making the correct choice. Although no non-responses were recorded in the cognitive flexibility phase of the task, a number of mice exhibited no choice per trial during initial acquisition of the OTOD (EE: *n* = 1, non-response: 1; SE: 12 mice, non-response (mean + standard error of the mean): 1.19 + 0.446). These non-responses were treated as missing values and excluded from GEE analysis.

To further explore the dynamics of OTOD task acquisition, RR and IDS, a change point analysis was performed on the cumulative record of correct responses for each animal during each task type (Gallistel et al., 2004). This assessment allowed for the detection of “change points” which marked the most dramatic variations in the slope of the cumulative record, a useful metric for identifying and defining changes in performance. We used a recursive algorithm (MATLAB functions provided by Gallistel et al., 2004, supporting information, *Proceedings of the National Academy of Sciences*, *USA* website: http://www.pnas.org; MATLAB, Mathworks, Natick, MA, USA) to search the individual cumulative records of performance (correct choice = 1, incorrect choice = 0) for putative change points (the trial where the record maximally deviated from a straight line drawn from the start of the record to each point in the record). A chi-square test was used to determine whether the frequency of correct responses before the putative change point significantly differed from that which followed it. A user specified “logit” value (log of the odds against the null hypothesis that there is no change; log[(1 − p)/p]) determined the strength of the evidence of a change in performance around the putative change point. If a change point passed the criteria set by the logit, the algorithm began the process again starting on the first post-change point trial. We ran the algorithm on each individual starting with the highest recommended logit value of 6 (corresponding to a *p*-value of 10^−7^) and progressing down in increments of 1 until we could detect at least one change point marking a statistically significant upward change in the slope of the cumulative record. Since a logit value of 1.28 is associated with a *p*-value of approximately 0.05, this was the lowest possible value used.

The number of trials to the first change point indicating an upward shift in performance (which provides a measure of how quickly the animals exhibited a significant improvement in performance) and the pre- (Phase 1) and post- (Phase 2) change point slopes of the cumulative record (the correct response rate for a given epoch and therefore providing an indication of initial as well as asymptotic (post change point to end of trial period) performance levels) were calculated for each individual.

This was executed on the record of all trials across the entire OTOD task acquisition as well as for each temporal distance separately. For RR and IDS profiles, the change point analysis was only executed on the entire record since there were too few trials to reliably detect change points if separated by distance. An independent samples *t*-test (or a Mann Whitney U-test where data were non-parametric, as indicated) was used to compare EE and SE groups on trials to first change point and a mixed-model ANOVA was used for pre- and post-change point slope comparisons (Prism, GraphPad, La Jolla, CA, USA).

## Results

### Enrichment Affects Overall OTOD Performance

**Figure 2:**
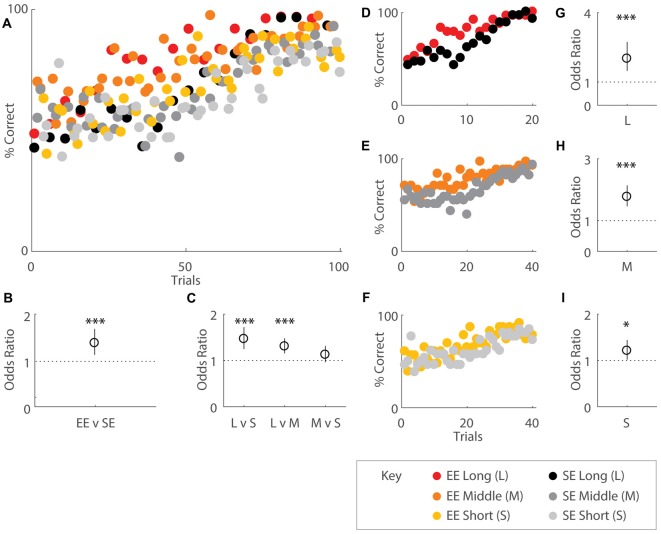
Environmental enrichment (EE) affects overall acquisition of the OTOD task. Performance indicated by percentage correct over total choices for groups of EE (coloured) and standard environment (SE; grey scale) mice, all distances combined and plotted in accordance to OTOD task training schedule **(A)** While both housing cohorts appeared to exhibit roughly random performance levels (50%) at the beginning, EE mice appeared to reach higher percentages earlier over the course of the training period than SE animals. **(B)** Odds ratio (OR) comparing performance by housing group. EE mice were roughly 1.5 times more likely to correctly choose the odor presented earlier in the sequence compared SE cohorts (OR = 1.49, *p* < 0.001). **(C)** OR comparing performance by temporal distances between odour choices. Mice were 1.46 times more likely to choose the correct odour for long (L) compared to short temporal spans (S) (L vs. S: OR = 1.46, *p* < 0.001) as well as 1.3 times more likely to make the appropriate choice in L vs. middle (M) level separations (L vs. M: OR = 1.30, *p* < 0.001). No difference was observed between M and S distances (*p* = 0.146). **(D–F)** Performance across the entire training period represented as percentage correct over total choices for EE and SE cohorts, separated by distance. Colour designations are identical to **(A)** above. OR comparing performance by housing group for L **(G)**, M **(H)**, and S **(I)** distances. Enriched mice were more ~2 times more likely to choose correctly for the longest (OR = 2.01, *p* < 0.001), 1.77 times more likely for middle (OR = 1.77, *p* < 0.001), and 1.21 times more likely for the shortest temporal separation (OR = 1.21, *p* = 0.034) between choice odours. Red: EE, long distance; orange: EE, middle distance; yellow: EE, short distance; black: SE, long distance; dark grey: SE, middle distance; light grey: SE, short distance (see key). *p < 0.05; ***p < 0.001.

Overall, both enriched and standard groups improved their performance over time for all three temporal separations tested, considered together or separately (Figure 2). Qualitative assessment of performance across trials (reflected in the percentage correct for all subjects within a given housing group at each trial; Figures 2A,D–F) suggested that initially reward was gained by chance (~50% correct). By the end of training however, both groups were able to achieve mean performance scores that were greater than 80%, indicating that the animals had successfully acquired the task.

Quantitative analysis revealed that EE mice exhibited higher performance scores compared to SE mice especially during earlier learning stages, suggesting that EE fosters improved task acquisition (Figures 2A,D–F). When performance was regressed on housing conditions and distances, enrichment was revealed to affect overall task acquisition (*b* = 0.398, s.e.r. = 0.0508, OR = 1.49, *p* < 0.001), with enriched animals roughly 1.5 times more likely to make correct choices overall (Figure 2B). Distances also had an effect, with significant differences observed between long and short (*b* = 0.381, s.e.r. = 0.0825, OR = 1.46, *p* < 0.001), long and middle (*b* = 0.265, s.e.r. = 0.0628, OR = 1.30, *p* < 0.001), but not middle and short (*b* = 0.116, s.e.r. = 0.0797, OR = 1.12, *p* = 0.146) temporal spans, consistent with the notion that the ability to distinguish events is facilitated with greater inter-presentation intervals for both groups (Figure 2C).

When temporal separations were considered separately, EE mice also exhibited improved performance compared to SE mice for each separation (long: *b* = 0.699, s.e.r. = 0.160, OR = 2.01, *p* < 0.001; Figures 2D,G; middle: *b* = 0.571, s.e.r. = 0.0970, OR = 1.77, *p* < 0.001; Figures 2E,H; short: *b* = 0.190, s.e.r. = 0.0894, OR = 1.21, *p* = 0.034; Figures 2F,I).

### OTOD Task Acquisition Is Accelerated in Enriched Mice

The acquisition of novel discrimination tasks through trial-and-error learning can often be characterised by discrete, detectable and often abrupt transitions in performance levels across trials (Gallistel et al., 2004; Durstewitz et al., 2010). In order to gain a deeper quantitative appreciation of the influence of EE on improving OTOD performance, a change point analysis was conducted on learning trajectories of individual animals (cumulative record as a function of decision trials) for all three distances tested, both together and separately.

**Figure 3:**
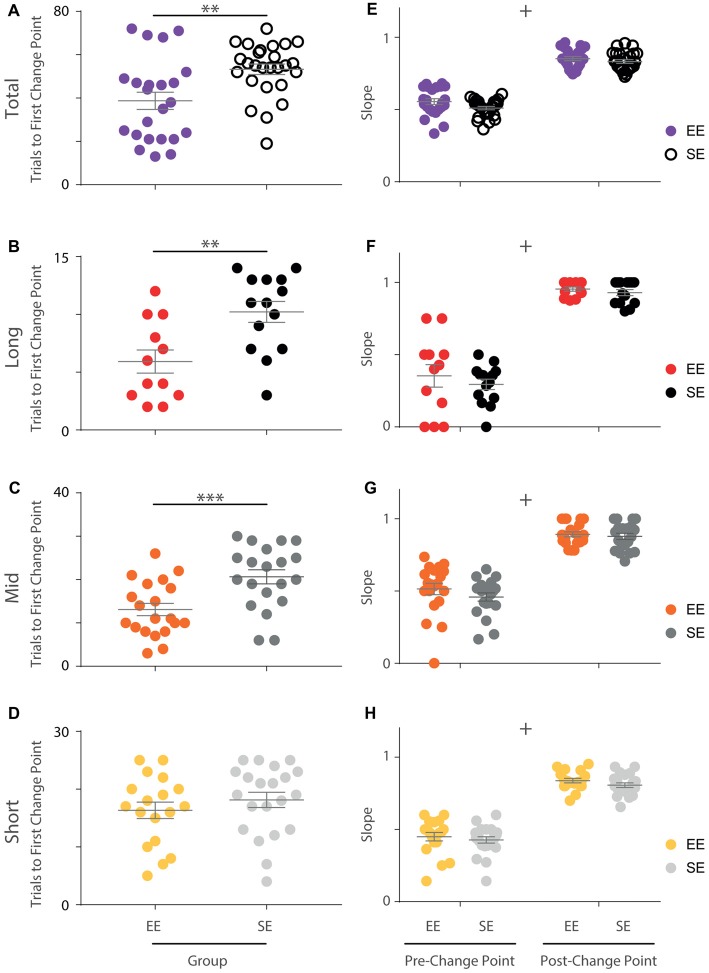
Initial OTOD acquisition is accelerated in EE mice. **(A)** The number of trials required by each individual to reach the first change point marking an upward transition in performance is significantly less for enriched compared to standard housed animals with all temporal distances combined (Mann Whitney U-Test, *U* = 157, *p* < 0.005). **(B–D)** EE vs. SE trial numbers to first performance change point separated by temporal distance between choice odours. Significantly fewer trials were required by EE mice for long (**B**; *t*_(24)_ = −3.20, *p* < 0.005) and middle (**C**; *t*_(38)_ = −3.51, *p* < 0.005), but not short (**D**; *t*_(37)_ = −0.930, *p* = 0.358) distances. **(E)** The slope of the cumulative record (i.e., correct response rate) before and after the first change point were not found to be significantly different between EE and SE cohorts when all temporal distances were considered together (group (*F*_(1,47)_ = 3.16, *p* = 0.082), phase (*F*_(1,47)_ = 896, *p* < 0.001), group × phase (*F*_(1,47)_ = 1.40, *p* = 0.242)). **(F–H)** Cumulative record slopes before and after the first change point for each animal, plotted separately by temporal distance. No significant differences between housing cohorts were detected (long: group (*F*_(1,24)_ = 0.800, *p* = 0.38), phase (*F*_(1,24)_ = 248.76, *p* < 0.001), group × phase (*F*_(1,24)_ = 0.191, *p* = 0.666); middle: group (*F*_(1,38)_ = 1.13, *p* = 0.294), phase (*F*_(1,38)_ = 329, *p* < 0.001), group × phase (*F*_(1,38)_ = 0.881, *p* = 0.35); short: group (*F*_(1,37)_ = 1.39, *p* = 0.246), phase (*F*_(1,37)_ = 386, *p* < 0.001), group × phase (*F*_(1,37)_ = 0.056, *p* = 0.814)). Colour designations indicated by key. **p < 0.005; ***p < 0.001; ^+^*p* < 0.001, phase only.

When examining the cumulative sum of correct responses across the entire testing period, all animals exhibited at least one statistically significant change point, which marked the trial on which the performance of the animal improved most dramatically. EE mice required fewer trials to reach the first change point, indicating an accelerated upward transition in performance (Mann Whitney U-Test, *U* = 157, *p* < 0.005; Figure 3A). There was no detectable difference in logit values used to detect at least one significant change point between EE and SE animals (independent samples *t*-test: *t*_(47)_ = 1, *p* > 0.3, logit range corresponded to approximate *p*-values ranging from 0.05 to 1.00 × 10^−6^ for both groups; data not shown).

When distances were considered separately only a portion of the animals exhibited a statistically significant change point (EE: 12/23 for long, 20/23 for middle, 18/23 for short; SE: 14/26 for long, 20/26 for middle, 21/26 for short). Nonetheless, when animals that demonstrated a change point were compared, upward transitions in performance occurred earlier for enriched animals compared to standard animals for long and middle, but not short distances (independent samples *t*-tests; long: *t*_(24)_ = −3.20, *p* < 0.005; middle: *t*_(38)_ = −3.51, *p* < 0.005; short: *t*_(37)_ = −0.930, *p* = 0.358; Figures 3B–D). For the animals included in this analysis, the same approximate logit value (corresponding to a *p*-value of ~0.05) was used for change point detection.

In order to explore these differences further, the slopes of the cumulative performance plots before (Phase 1) and after (Phase 2) the identified change point were calculated. While the slopes were measurably different between the two phases, no housing effect was detected either for all trials together (Phase 1: EE = 0.55; SE = 0.51. Phase 2: EE = 0.85; SE = 0.83; mixed-model ANOVA: group (*F*_(1,47)_ = 3.16, *p* = 0.082), phase (*F*_(1,47)_ = 896, *p* < 0.001), group × phase (*F*_(1,47)_ = 1.40, *p* = 0.242); Figure 3E) or when considered separately by distance (mixed-model ANOVAs; long: group (*F*_(1,24)_ = 0.800, *p* = 0.38), phase (*F*_(1,24)_ = 249, *p* < 0.001), group × phase (*F*_(1,24)_ = 0.191, *p* = 0.666); middle: group (*F*_(1,38)_ = 1.13, *p* = 0.294), phase (*F*_(1,38)_ = 329, *p* < 0.001), group × phase (*F*_(1,38)_ = 0.881, *p* = 0.354); short: group (*F*_(1,37)_ = 1.4, *p* = 0.246), phase (*F*_(1,37)_ = 386, *p* < 0.001), group × phase (*F*_(1,37)_ = 0.056, *p* = 0.814); Figures 3F–H), suggesting that the rate of acquisition before and after performance change points was not affected by enrichment.

Together these findings suggest that although both groups acquired and performed the task in a similar fashion, EE animals acquired the task more rapidly than their SE counterparts.

### Enrichment Affects Immediate Performance After Both Intra-Dimensional Shifts and Rule Reversals

Survival is dependent on an animal’s ability to adapt to contingencies resulting from a rapidly and continuously changing environment. In an attempt to gain an overall impression of how enrichment can influence behaviour flexibility, the responses of mice from SE and EE cohorts trained on the OTOD were compared on one of two new conditions: (1) an inversion of the learned rule (Rule Reversal; RR) in which subjects were exposed to the original group of five odours presented in different sequences at the beginning of each trial as before, but were then required to identify the most recently presented scent to gain reward (SE: *n* = 6; EE: *n* = 7); or (2) an IDS condition, in which mice had to apply the original learned rule to an unfamiliar set of five exemplar odours (SE: *n* = 6; EE: *n* = 7).

**Figure 4:**
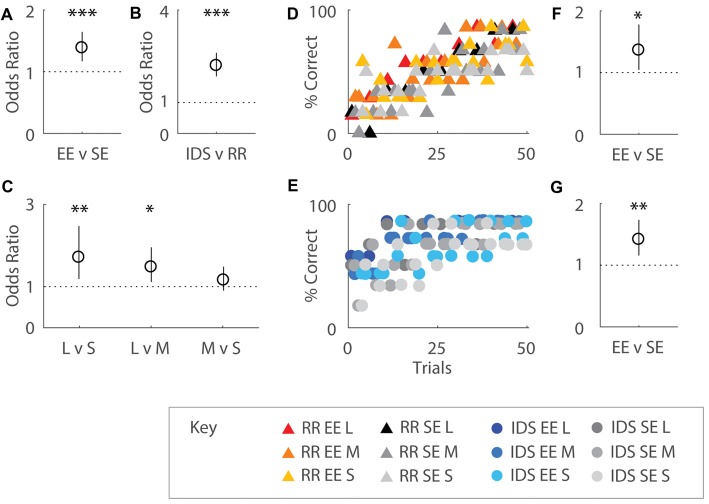
Enrichment affects immediate performance after both Intra-Dimensional Shifts (IDSs) and Rule Reversals (RR). **(A)** Odds ratio (OR) comparing performance by housing group over 50 trials. EE mice were ~1.4 times more likely to correctly choose the odor presented earlier in the sequence compared SE cohorts (OR = 1.39, *p* < 0.001). **(B)** OR comparing performance by task transition type. Mice allocated to the IDS task were 2.20 times more likely to respond correctly compared to cohorts assigned to the RR version of the OTOD task (OR = 2.20, *p* < 0.001). **(C)** OR comparing performance by temporal distances between odour choices on post-transition versions of the task. Significant differences were detected between different presentation spans, with 1.71 times better choice selection observed for long compared to short (OR = 1.71, *p* = 0.004) and 1.48 times for long over middle (OR = 1.48, *p* = 0.007) distances. No difference between middle and short spans was detected (*p* = 0.24). **(D,E)** Performance across training period represented as percentage correct over total choices for EE and SE cohorts, separated by post-transition task: RR **(D)** and IDS **(E)**. SE and EE cohorts initially exhibited poor performance in the RR OTOD, indicating a perseverant usage of obsolete rules. Percentage correct for both groups dropped to chance levels immediately after change was implemented in the IDS version of the task. OR comparing performance by housing group for RR **(F)** and IDS **(G)** versions of the task. Enriched mice were more 1.36 times more likely to choose correctly in the RR version compared to SE cohorts (OR = 1.36, *p* = 0.022). Similarly EE animals were 1.42 times more likely than SE mice to make the correct choice in the IDS OTOD (OR = 1.42, *p* = 0.001). Colour designations indicated by key. *p < 0.05; **p < 0.005; ***p < 0.001.

Qualitative analysis revealed a dramatic difference in initial performance between the two new tasks. When examining performance for all subjects in each group, mice that underwent RR persevered with the previously learned obsolete rule during the initial stages of task re-acquisition (illustrated by “below chance” (~20%) mean performance levels exhibited by both housing groups across the first few trials; Figure 4D). Improved choice selection over the brief training period, however, suggests that the mice rapidly adapted to the change in contingencies. Animals undergoing the IDS task exhibited initial choice behaviour that allowed “chance” level access to reward (i.e., ~50% correct). As the number of trials progressed, these animals also exhibited clear signs of performance improvement (Figure 4E).

When performance was regressed on housing conditions, task type, and temporal distances, all three factors were shown to exert an influence on choice selection. Overall, EE mice were ~1.4 times more likely to choose the correct odour compared to SE cohorts (*b* = 0.326, s.e.r. = 0.0870, OR = 1.39, *p* < 0.001; Figure 4A), suggesting that enriched animals exhibited greater adaptability to changing conditions. Performance on IDS was also almost 2.2 fold better than the RR version of the task (*b* = 0.788, s.e.r. = 0.0878, OR = 2.20, *p* < 0.001; Figure 4B) for all mice, presumably due to perseverance of initial choice behaviours immediately after contingency reversal for the latter. An effect of temporal separation on performance was also observed (Figure 4C), with better choice selection for long compared to short (*b* = 0.538, s.e.r. = 0.188, OR = 1.71, *p* = 0.004) and middle (*b* = 0.390, s.e.r. = 0.144, OR = 1.48, *p* = 0.007) distances (no difference between middle and short was detected: *b* = 0.148, s.e.r. = 0.127, OR = 1.16, *p* = 0.244), similar to that which was observed during initial acquisition.

When considered separately by task type, EE mice exhibited better recovery for both task variations. Enriched mice were more 1.36 times more likely to choose correctly in the RR version compared to SE cohorts (RR: *b* = 0.310, s.e.r. = 0.136, OR = 1.36, *p* = 0.022; Figure 4F). Similarly EE animals were 1.42 times more likely than SE mice to make the correct choice in the IDS OTOD task (IDS: *b* = 0.349, s.e.r. = 0.103, OR = 1.42, *p* = 0.001; Figure 4G). Temporal distance, only affected the IDS cohort (Long vs. Short: *b* = 1.021, s.e.r. = 0.278, OR = 2.78, *p* < 0.001; Long vs. Middle: *b* = 0.678, s.e.r. = 0.195, OR = 1.97, *p* = 0.001; Middle vs. Short: *b* = 0.343, s.e.r. = 0.230, OR = 1.41, *p* = 0.136; not shown).

Together, these findings suggest that EE can instil advantages in choice selection even under conditions of uncertainty.

### Enrichment Accelerates Performance Recovery in Intra-Dimensional Shifting But Not Rule Reversal Conditions

In order to better characterise the manner in which EE affected OTOD performance after a change in task contingencies, change point analyses were conducted on learning trajectories of individual animals for both RR and IDS cohorts.

**Figure 5:**
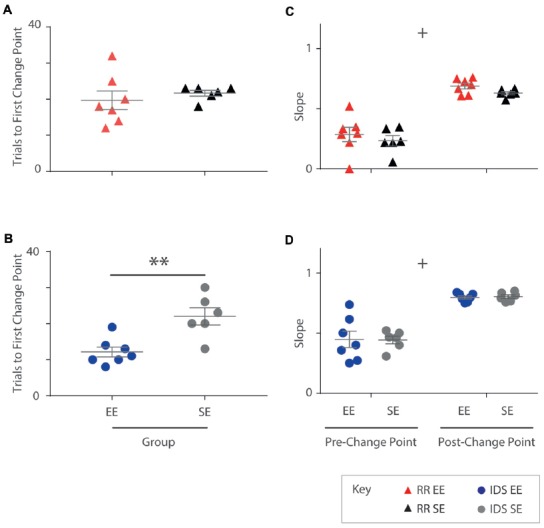
Enrichment accelerates performance recovery after an IDS but not a Rule Reversal. **(A)** The number of trials required by each individual to reach the first change point marking an upward transition in performance was not different for EE vs. SE animals for the Rule Reversal (RR) task (Mann-Whitney U test: *U* = 13.5, *p* = 0.295). **(B)** An upward shift in performance during the IDS, however, was detected after fewer trials in enriched animals when compared to standard housed animals with all temporal distances combined (independent samples *t*-test: *t*_(11)_ = −3.73, *p* < 0.005). **(C,D)** The slope of the cumulative record (i.e., correct response rate) before and after the first change point were not found to be significantly different between EE and SE cohorts either for the RR (C; group (*F*_(1,11)_ = 1.44, *p* = 0.256), phase (*F*_(1,11)_ = 149, *p* < 0.001), group × phase (*F*_(1,11)_ = 0.011, *p* = 0.920), or IDS versions of the task [**D**; group (*F*_(1,11)_ = 0, *p* = 0.990), phase (*F*_(1,11)_ = 86.9, *p* < 0.001), group × phase (*F*_(1,11)_ = 0.0182, *p* = 0.896)]. **p < 0.005; ^+^*p* < 0.001, phase only.

All subjects exhibited at least one statistically significant upward transition (Figure 5). In the RR version of the task, no differences in trial number to the first change point were detected between housing cohorts (Mann-Whitney U test: *U* = 13.5, *p* = 0.295; Figure 5A). For the IDS task, however, EE mice exhibited an upward performance shift in significantly fewer trials compared to SE animals (independent samples *t*-test: *t*_(11)_ = −3.73, *p* < 0.005; Figure 5B). For these analyses, the same approximate logit value (corresponding to a *p*-value of ~0.05) was used for change point detection in all animals.

No differences between housing groups were detected in performance slopes before and after the first upward change point for either the RR (Phase 1: EE = 0.29; SE = 0.23. Phase 2: EE = 0.69; SE = 0.63; repeated measures ANOVA: group (*F*_(1,11)_ = 1.44, *p* = 0.256), phase (*F*_(1,11)_ = 149, *p* < 0.001), group × phase (*F*_(1,11)_ = 0.011, *p* = 0.920); Figure 5C) or IDS versions of the task (Phase 1: EE = 0.45; SE = 0.44. Phase 2: EE = 0.80; SE = 0.80; repeated measures ANOVA: group (*F*_(1,11)_ = 0, *p* = 0.990), phase (*F*_(1,11)_ = 86.9, *p* < 0.001), group × phase (*F*_(1,11)_ = 0.0182, *p* = 0.896); Figure 5D), despite measureable changes between phases, suggesting again that the influence of enrichment was limited within each of these learning epochs.

These findings suggest that for the IDS task, performance improvements observed in EE mice were due to an expedited adjustment in behavioural strategy, manifesting as an application of the previously learned rule to a new set of exemplar stimuli.

## Discussion

The successful performance of the OTOD task is thought to require the processing of memory for temporal order or relative recency, which is mediated by the hippocampus (Fortin et al., 2002; Tse et al., 2011; Albasser et al., 2012). Initial analysis of the present data revealed that EE mice exhibited enhanced performance on this OTOD task, which could be interpreted as improved memory processing for temporal order of olfactory stimuli. Closer examination of individual performance using a change point analysis, however, showed that, once the task was acquired, EE and SE cohorts were able to execute the OTOD task with the same accuracy at all temporal distances tested, suggesting that the ability to temporally resolve events was not dramatically affected. Crucially, our analysis demonstrates that EE mice learned the task significantly faster than their SE counterparts.

Although the control housing condition is referred to here, and in the bulk of the relevant literature as “standard”, it should be noted that conventional laboratory housing conditions can be considered to provide a somewhat deprived sensory experience relative to what would be encountered by mice that are not held in captivity. Accordingly, the EE paradigm used in this study may be providing a more naturalistic environment for pups to develop (Arai et al., 2009). While future work will be required to determine the degree to which EE in laboratory settings emulates real-world engagement, our findings do provide an indication of how enhancing the day-to-day experience of developing mice can influence their acquisition and performance on complex cognitive tasks relative to pups raised in SE housing conditions.

The mice in this study began their training while they were at a relatively immature stage, P30. Both the striatum and hippocampus, structures important for the acquisition and execution of the OTOD, begin to form perineuronal nets, extracellular structures associated with network consolidation and associated with the closing of heightened periods of neural plasticity during early development (critical periods), at roughly 3 weeks after birth (Brückner et al., 2000; Lee et al., 2008). Thus, this stage was chosen to elucidate the effect of EE on the emergence of complex cognitive function potentially related to the consolidation of circuits in these vital areas.

As task acquisition continued for 2 months from around P30, training over this period in and of itself could have potentially provided enriching experiences for both EE and SE mice. Despite this, possible confound, we nevertheless observed a difference in acquisition during task engagement based on housing conditions. Indeed, it is possible that further changes due to EE from birth may have been masked due to the enriching effects of prolonged training in SE mice. Whether the observed, accelerated learning was due to a cumulative effect of enrichment from birth overlapping with task related training, or solely a result of early enrichment is not clear. Moreover, as training was initiated in adolescents, whether the effects we observed would be detectable in mice trained as adults is not known. Further experiments separating and isolating early enrichment from task acquisition in mature animals will be required to determine whether the observed differences in the current study can be uniquely attributable to the early enrichment period.

Given that mice have an innate preference for exploration of novelty (Smith et al., 2009; Ennaceur, 2010), it is conceivable that a bias for odours experienced less recently (rather than temporal recall *per se*) could have successfully engendered above-chance performance in the OTOD task, which required the animal to choose the odour experienced earlier in the sequence. The observation that both housing cohorts exhibited chance-level performance in the early stages of task acquisition, however, suggests that innate novelty seeking drive did not explicitly instruct choice behaviour from the outset of training. While this natural proclivity may well have been utilised by the animals in order to perform the task, the strategy used for successful completion still had to be learned.

Although this study does not provide any direct evidence for the mechanisms underlying the improved performance observed in EE mice, it is possible that enrichment enhanced one of a number of sensory, learning and memory-associated, and decision-making processes to facilitate task acquisition. First, an enhanced ability to identify discrete odours may have facilitated OTOD learning. EE has been shown to improve performance on olfactory discrimination tasks (Mandairon et al., 2006c), as well as short-term olfactory memory (Rochefort et al., 2002; Veyrac et al., 2009). The inclusion of additional odours in the home-cage of EE mice, while different from the cues used in OTOD training, may nevertheless have further contributed to enhancing olfactory sensitivity in these animals. Our observation that performance at the end of the training period (asymptotic performance), another metric associated with improved sensory function (Cancedda et al., 2004), did not differ between housing cohorts (as revealed/confirmed by our change point analysis), however, suggests that improved sensory processing, while critical, may have only partially contributed to task acquisition and performance in EE mice.

A number of mnemonic processes may also have been employed by the animals (such as relative recency, novelty/familiarity detection, episodic-like recall etc.) to learn and execute OTOD. Several lines of evidence have shown that spatial pattern separation is improved by enrichment and related processes (Creer et al., 2010; Sahay et al., 2011), which if applied to the current temporal context, may allow for each odour (episode) within a given sequence to be encoded more discretely by enriched animals. Novelty/familiarity or recency coding, a mechanism by which rodents have been explicitly shown to differentiate events that have occurred in the past, can also be rescued by EE in mice suffering from traumatic brain injury (Darwish et al., 2014). Regardless of how the animals were solving the task, even slight improvements in mnemonic accuracy due to enrichment may have expedited the identification of rules relevant to successful task completion.

Successful execution of the task must have also engaged processes that link this relevant information to a course of actions that would lead to positive outcomes. The mapping of such associations involves the activation of a number of interacting brain regions, including the hippocampal formation (Fortin et al., 2002), striatum (Sleezer et al., 2016), as well as medial and orbital prefrontal areas (Wallis et al., 2001; Tse et al., 2011) ultimately contributing to the formation of contextually relevant rules, cognitive sets, schemas and/or maps (Block et al., 2007; Tse et al., 2011; Kosaki and Watanabe, 2012; Wikenheiser and Schoenbaum, 2016; Armelin et al., 2017). As EE has been proposed to facilitate functional connectivity between cortical and subcortical areas (Spires et al., 2004), it is possible that similar mechanisms may have improved the ability of enriched mice to rapidly link cue-action-outcome associations necessary to acquire and perform the OTOD task.

Further, establishing appropriate rules of engagement for a particular task is potentially a dissociable process from being able to use this information to make appropriate decisions or execute a correct set of actions (Tsujimoto et al., 2011). Given the reported improvement in reversal learning exhibited by enriched mice (Zeleznikow-Johnston et al., 2017), a function dependent on orbitofrontal cortex (Schoenbaum et al., 2003; Kim and Ragozzino, 2005; Marquardt et al., 2017), it is also possible that EE may facilitate the accessibility of relevant and appropriate rules or contextual information thus making them more likely to influence choice preferences and actions, again potentially contributing to expedited acquisition of the odour sequencing task.

The responses exhibited to the two modified versions of the OTOD task provides some insight into the degree to which some of these processes may have been differentially affected by EE. In the IDS version of the task, a single contingency change was implemented: a specific feature of the stimulus (i.e., odours) was changed while the rules governing successful completion of the task were maintained. EE mice exhibited expedited performance recovery under these conditions, even though initial, post-transition performance fell to chance levels. This suggests again that mice of both housing groups did not immediately utilise any innate preference for novelty, echoing the lack of novelty bias observed in the early stages of OTOD acquisition.

While the assignment of new olfactory exemplars in this task has been nominally identified as an “IDS”, as only one contingency change was tested in animals initially trained on a single set of odours, the experience of the subjects in this context would have been akin to the formation of a novel stimulus-reward association (as supported by the return of mean performance to chance levels). In light of this, the accelerated performance recovery as revealed by the change point analysis for EE mice may reflect an enhanced ability to update the cue-reward association to encompass the new task-related olfactory exemplars, possibly via enhanced medial prefrontal function (Tse et al., 2011). Further studies will be required to clarify the contribution of each of these processes.

Conversely, in the rule reversal condition, while EE cohorts exhibited improved overall choice selection, consistent with previously reported changes in reversal learning for enriched mice (Zeleznikow-Johnston et al., 2017), a change point analysis did not reveal any significant housing group differences in terms of performance recovery. Mice engaged in RR tasks often exhibit a perseverance effect, maintaining the use of previously rewarded cue-action associations immediately after a change in contingencies have been implemented (Ragozzino, 2007; Marquardt et al., 2017). Although individual performance varied considerably, the fact that the number of trials to the first upward performance transition did not differ between housing cohorts, suggests that the ability to initially switch choice behaviour (i.e., the degree of perseverance), at least after a single reversal, is not overtly affected by enrichment.

The overall change in performance observed EE mice may instead reflect an improved ability to form new cue-action-outcome associations, consistent with augmented sensory/mnemonic processing implicated in the IDS results, a process that would have been initiated after abandoning the original rule. While the limited number of post-transition trials did not permit a closer examination of individual performance dynamics after the first upward transition, future studies should endeavour to explicitly examine how EE affects a subject’s ability to consolidate newly learnt associations upon discarding previously acquired knowledge; key processes that contribute to behavioural flexibility.

In conclusion, EE expedited the ability of mice to discriminate the temporal order of olfactory stimuli. This improvement appears to be related primarily to an earlier manifestation of learning on choice behaviour. EE animals also exhibited generally enhanced performance after a rule reversal, consistent with previous findings (Zeleznikow-Johnston et al., 2017), and specifically accelerated performance recovery after an intra-dimensional-like shift in task contingencies. To the best of our knowledge, this is the first demonstration of EE’s influence on a task that engages multiple, explicitly definable memory systems. Together, these findings contribute to the growing body of work that emphasises the profound impact environmental conditions during early development can have on processes associated with learning and flexible behaviour, critical for survival throughout an animal’s life.

## Author Contributions

DR-H acquired the data. DR-H, TJB, CAL and AS contributed to the design of the work and interpretation of the data. TJB and AS performed the bulk of the analysis. DR-H, TJB and CAL contributed to the writing and editing of the manuscript. AS was responsible for writing, compiling and editing the final draft of the manuscript.

## Conflict of Interest Statement

The authors declare that the research was conducted in the absence of any commercial or financial relationships that could be construed as a potential conflict of interest.
